# Release kinetics of cardiac biomarkers in patients undergoing valve replacement surgery for rheumatic heart disease

**DOI:** 10.1186/1749-8090-8-S1-P145

**Published:** 2013-09-11

**Authors:** SK Agarwal, S Singh, A Kapoor, S Pandey, A Sinha, S Kumar, H Rai, S Tewari, N Garg, PK Goel

**Affiliations:** 1 Cardio-Thoracic Surgery, Sanjay Gandhi PGIMS, Lucknow, India; 2 Cardiology, Sanjay Gandhi PGIMS, Lucknow, India

## Background

Levels of brain natriuretic peptide (BNP) increase following CABG and predict post-operative outcomes. Release kinetics of BNP, Troponin-I (TnI) and CKMB after valve replacement are not well characterized.

## Methods

We assessed levels of these biomarkers 24 hours prior and 6,24, 48 hrs, 1 month following mitral/aortic valve replacement in 50 patients (mean age 36.7 yrs, LVEF 54.4%, 80% males).

## Results

Mean baseline BNP, TnI and CK-MB levels were 304.01 pg/ml, 0.03 ng/ml and 0.99 ng/ml. BNP initially decreased within 6 hours of surgery, and peaked at 24 hours; TnI and CKMB showed an early rise, with declining trends by 24 hrs. Peak BNP levels occurred in 90% patients by 24-48 hrs, while for TnI and CKMB this occurred in only 15-30%.Mean delta (peak-baseline) BNP, TnI, CKMB was 660.1pg/ml, 8.1ng/ml and 32.3ng/ml.At 1 month, levels of all biomarkers were not significantly different from baseline. Patients with higher baseline BNP more commonly had atrial fibrillation (71vs 33%,p=.02), higher right ventricular systolic pressure (69.7vs43.9 mm Hg,p< 0.001),higher Euroscore II(2.42vs1.49,p=0.006),longer inotrope duration (56.1vs26.5hrs,p=0.03), ventilator support time (35.6vs21.7 hrs,p=0.04), longer ICU (4.8vs3.2 days,p=0.02) and hospital stay (6.8vs5.2 days,p=0.03). Inotrope duration>42 hrs, ventilation time>29 hrs and ICU stay>4 days was seen in 42%vs19%, 30%vs9% and 33%vs14% respectively in patients with baseline BNP >/< 200 pg/ml. Only baseline BNP was a significant predictor of inotrope duration (p=0.01) and ventilation time (p=0.02). Only 24 hour post-operative BNP and delta BNP were predictors of inotrope duration>42 hrs, ventilation time>29 hrs and ICU stay>4 days.

## Conclusion

Release kinetics of cardiac biomarkers following valve surgery are significantly different from each other. Of all the biomarkers, only BNP levels had an association with post-operative inotrope duration, ventilation time and ICU stay in patients undergoing valve replacement.

